# Comparing sterile male releases and other methods for integrated control of the tiger mosquito in temperate and tropical climates

**DOI:** 10.1038/s41598-021-86798-8

**Published:** 2021-04-01

**Authors:** Léa Douchet, Marion Haramboure, Thierry Baldet, Gregory L’Ambert, David Damiens, Louis Clément Gouagna, Jeremy Bouyer, Pierrick Labbé, Annelise Tran

**Affiliations:** 1 CIRAD, UMR ASTRE, 97491 Sainte-Clotilde, Reunion France; 2 ASTRE, CIRAD, INRAE, Univ Montpellier, Montpellier, France; 3 ISEM, CNRS, IRD, EPHE, Université de Montpellier, Montpellier, France; 4 TETIS, AgroParisTech, CIRAD, CNRS, INRAE, Univ Montpellier, Montpellier, France; 5 Department of Research and Development, EID Méditerranée, Montpellier, France; 6 IRD, CNRS-UM-IRD, UMR MIVEGEC, Montpellier, Reunion France; 7 IRD/GIP CYROI, Sainte-Clotilde, Reunion France; 8 CIRAD, UMR ASTRE, 34398 Montpellier, France; 9 Insect Pest Control Laboratory, Joint FAO/IAEA Programme of Nuclear Techniques in Food and Agriculture, 1400 Vienna, Austria; 10 CIRAD, UMR ASTRE, 97410 Saint-Pierre, Reunion France

**Keywords:** Invasive species, Ecological modelling, Population dynamics

## Abstract

The expansion of mosquito species worldwide is creating a powerful network for the spread of arboviruses. In addition to the destruction of breeding sites (prevention) and mass trapping, methods based on the sterile insect technique (SIT), the autodissemination of pyriproxyfen (ADT), and a fusion of elements from both of these known as boosted SIT (BSIT), are being developed to meet the urgent need for effective vector control. However, the comparative potential of these methods has yet to be explored in different environments. This is needed to propose and integrate informed guidelines into sustainable mosquito management plans. We extended a weather-dependent model of *Aedes albopictus* population dynamics to assess the effectiveness of these different vector control methods, alone or in combination, in a tropical (Reunion island, southwest Indian Ocean) and a temperate (Montpellier area, southern France) climate. Our results confirm the potential efficiency of SIT in temperate climates when performed early in the year (mid-March for northern hemisphere). In such a climate, the timing of the vector control action was the key factor in its success. In tropical climates, the potential of the combination of methods becomes more relevant. BSIT and the combination of ADT with SIT were twice as effective compared to the use of SIT alone.

## Introduction

Native to Asia^1^, the tiger mosquito *Aedes albopictus* (Skuse, 1894) has colonized America, Africa and Europe along with the intensification of globalization^2–4^. Its great ecological plasticity, due to specific traits such as its ability to colonize a wide range of larval sites and to feed on a wide variety of hosts, its diapause capacity, and the tolerance of its eggs to desiccation^5,6^, has enabled this spectacular worldwide establishment. The species has become established on every continent, from tropical to temperate regions^7^. A vector of dengue, Chikungunya and Zika viruses, *Ae. albopictus* represents a major threat to human health^8–11^ and has been involved in numerous epidemics due to these viruses in tropical areas^12–15^. Although these viruses are not yet established in Europe, their frequent introduction by infected travellers (*e.g.*, Chikungunya in Italy, 2007^16^ and 2017^17^) increases the risk of outbreaks in regions where *Ae. albopictus* is abundant^17–20^. As there are no effective vaccines against these vector-borne diseases^21,22^, vector control remains the cornerstone of disease prevention.

*Aedes albopictus is adapted to urban areas, breeding in the numerous small containers filled with water and available around houses.* Insecticide spraying and the mechanical destruction of potential breeding sites constitute the classic solutions to control outbreaks^23^. However, the behaviour of *Ae. albopictus*, which breeds in multiple cryptic and dispersed sites (tires, beverage cans, plastic items, etc...), hampers the effectiveness of these methods^24^. They therefore need to be supplemented to achieve sustainable control^25^.

Mass trapping and the autodissemination technique (ADT) are alternative control methods that are based on the behaviour of female mosquitoes^25,26^. Mass trapping consists of capturing females with artificial ovipositing sites (or ovitraps)^27–29^ or traps that mimic the presence of a blood-feeding source (Biogents Sentinel, BGS)^30,31^; the traps also capture males in search of a mate^32,33^. To overcome the difficulties of conventional insecticide-based methods to reach cryptic habitats, ADT uses the ovipositing behaviour of females to deliver the lethal agent: female mosquitoes are attracted to artificial breeding sites (stations) impregnated with a biocide, which they then transfer to natural breeding sites^34^. Both methods have shown promising reductions in mosquito populations^35–37^, but their efficiency relies heavily on the attractiveness and the density of traps and ADT stations^26,37,38^.

Another alternative for the control of *Aedes* populations is the sterile insect technique (SIT), which relies on the mass-release of males sterilized by ionizing radiation^25^. As females generally mate only once at the beginning of their lives, those that mate with sterilized males produce non-viable eggs, causing the target population to decline^25,39,40^. Significant reductions have been achieved in Italy^41^ and in China^42^. However, since the processes involved in producing large numbers of sterile males (mass rearing, handling and irradiation) may reduce their sexual performance^43^, the number of sterile males must be much higher than that of wild males for SIT to be effective, constituting a significant hindrance for large-scale application^39,40,44–46^. Furthermore, a very high rate of reduction in population density of *Ae. albopictus* populations is necessary to block the virus transmission^47^. A modified version of SIT known as boosted SIT (BSIT), which combines elements of SIT and ADT, has recently been proposed^48,49^. Released sterile males are coated with pyriproxyfen (PP), a biocide that inhibits the emergence of pupae^50,51^. PP can be transferred during mating to females which then, in turn, contaminate their breeding sites. However, BSIT remains in the experimental phase for the time being.

Due to the diversity of approaches, target species ecological contexts and logistical constraints, it is difficult to directly assess in the field the effect of each of these different techniques used alone, and even more so in combination. In such situations, mathematical models are useful tools that can provide insight into the ecological response to different mosquito population management strategies, and can help plan field trials (*eg*^52–59^). Several models have been developed to predict and understand the potential effects of SIT on mosquito populations^57,60–70^, and two recent studies have assessed the potential impact of BSIT. Pleydell *et al.* compared BSIT, SIT, and ADT in a constant environment^47^, while Haramboure *et al.* compared BSIT and SIT in realistic tropical ecological settings using a weather-driven mosquito population dynamics model^71^. Both studies concluded that BSIT would require fewer released sterile males, or could tolerate irradiated males with lower competitiveness, compared to SIT. However, to our knowledge, neither study used such models to compare all of the different control methods available, including conventional insecticide-based methods and their combinations.

The objective of the present study was therefore to take advantage of the weather-driven abundance model developed by Haramboure *et al.*^71^ to combine and compare different control methods against *Ae. albopictus* in realistic tropical and temperate climates. We extended this model, originally developed for a tropical area (Reunion Island, Indian Ocean), to the specificities (*e.g.*, winter diapause) of a temperate area, Montpellier (France), where *Ae. albopictus* has been established since 2010^72^. It should be noted that cases of Chikungunya transmitted locally by *Ae. albopictus* occurred in this city in 2014^73^. After validating the model accuracy on entomological data from an *Ae. albopictus* population without vector control, we performed a global sensitivity analysis to identify the key parameters affecting the impact of SIT and BSIT in temperate versus tropical climates. We also integrated the effect of prevention (*i.e.*, mechanical destruction of potential breeding sites), mass trapping (ovitraps or BGS-traps) and ADT stations on *Ae. albopictus* populations. Simulations were used to assess the effects of these different control methods, independently or in synergy with SIT and BSIT. This model thus provides a comprehensive evaluation of current vector control methods against the tiger mosquito, and can help control agencies plan their mosquito management strategies in different environments.

## Results

### Sterile male releases are the most effective control methods

**Figure 1:**
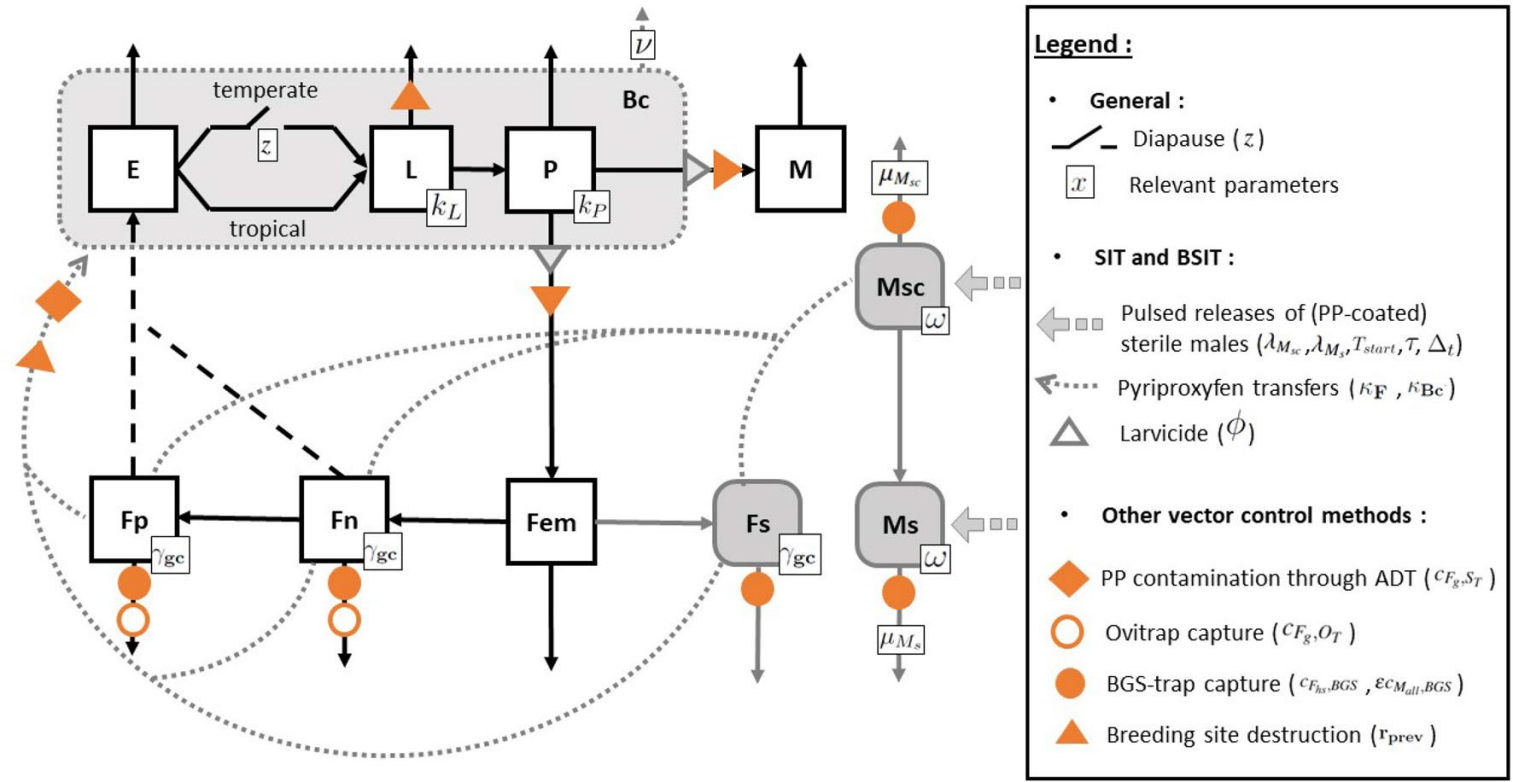
Simplified diagram of the model. The *Aedes albopictus* life cycle is computed in 7 stages: 3 are aquatic stages present in the breeding sites, eggs (*E*), larvae (*L*) and pupae (*P*), 4 are adult aerial stages, males (*M*), emerging females ($F_{em}$), nulliparous females ($F_n$) and parous females ($F_p$). Black arrows indicate transitions between stages. Diapause only occurs in the temperate climate and depends on the *z* parameter. Changes resulting from SIT and BSIT are indicated by grey lines and boxes representing sterile males, whether PP-coated ($M_{sc}$) or not ($M_s$), sterile females ($F_s$) and contaminated breeding sites ($B_c$). The key parameters, in particular those affected by vector control actions, are: $k_L$ and $k_P$ respectively the larval and pupae carrying capacities, $\gamma _{gc}$ the duration of the gonotrophic cycle, $\omega$ the relative competitiveness of sterile males, $\mu _{M_{sc}}$ and $\mu _{M_s}$ the mortality of sterile males, respectively PP-coated or not, $\nu$ the breeding site PP decontamination rate, and $\phi$ the probability for PP-exposed larvae to survive and pupate. Additional vector control actions were added to the model (orange): mass trapping (full circles for BGS-traps and hollow circles for ovitraps) according to the probability of capture (respectively $c_{F_{hs},BGS}, \epsilon c_{M_{all},BGS}$ and $c_{F_g,O_T}$), prevention (triangles) by reduction of breeding sites ($r_{prev}$), and PP autodissemination (diamonds for ADT) which depends on females contamination ($c_{F_g, S}$).

The weather-driven abundance model developed by Haramboure *et al.*^71^ in the context of the tropical climate of Reunion Island (Indian Ocean), and which already implements SIT and BSIT, was modified to (1) adapt it to a temperate climate by taking into account the winter season in Europe, with a diapause phase, and by modifying the values of the parameters to those observed in a temperate climate^74^, and (2) implement other vector control methods (Fig. 1): (a) prevention, through the destruction of breeding sites (triangles), (b) ovitraps (hollow circles) which capture only females, (c) BGS-traps (full circles) which capture all adults, and (d) ADT (diamonds) which contaminate the breeding sites (for more details see “Methods”). We then assessed the effects of the different control methods and their combinations by measuring the induced reduction rate, *i.e.*, the maximum reduction of fertilized females compared to an untreated population, and the resilience, *i.e.*, the time required for the population to recover similar dynamics to that of the untreated one.

**Figure 2:**
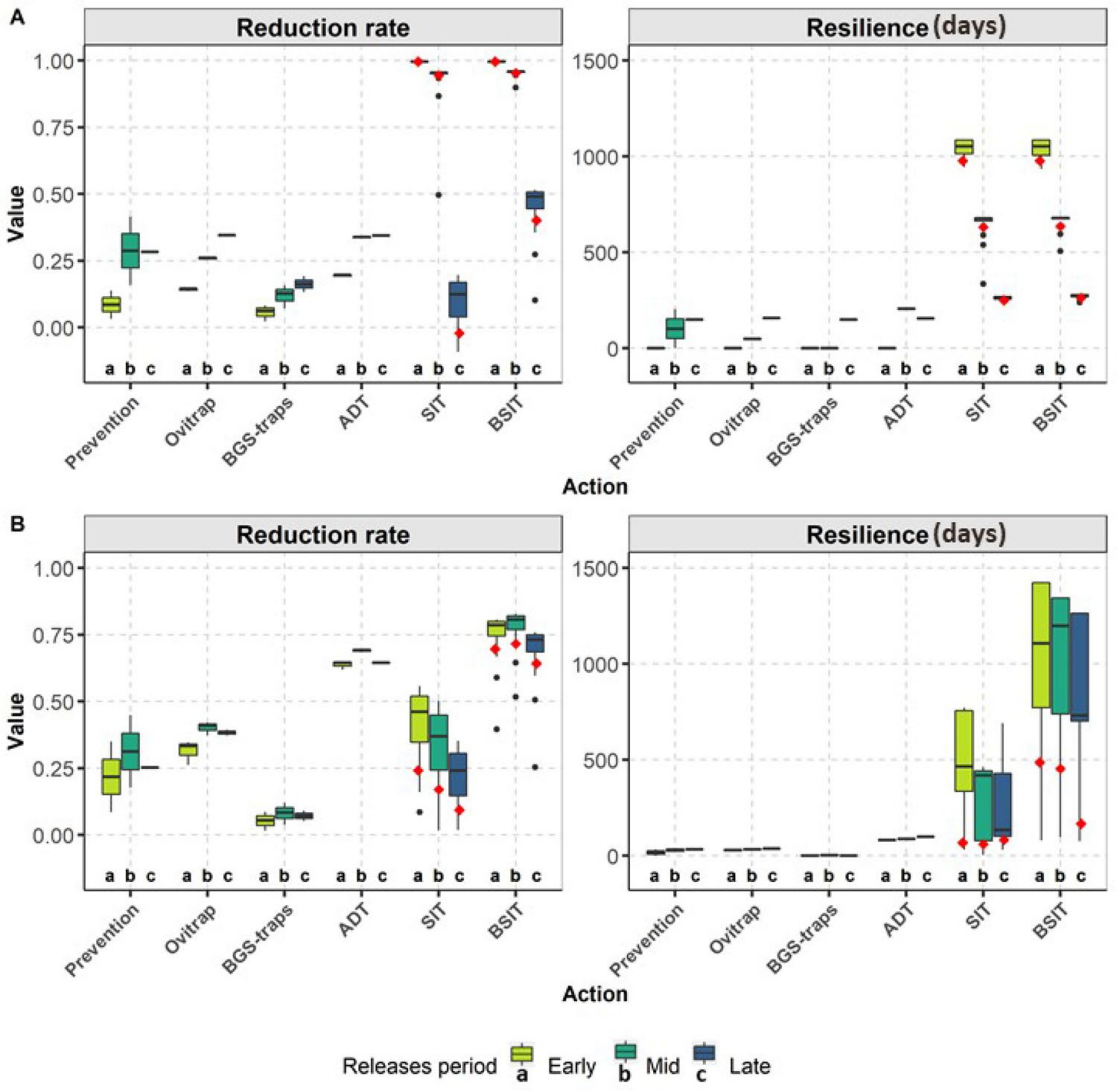
Reduction rate and resilience of vector control actions against *Aedes albopictus* in (**A**) a temperate climate and (**B**) a tropical climate. The boxplots show the outputs distribution for a range of efforts invested in vector control action (Table 1), i.e. the number of devices deployed in the area (ADT, ovitraps and BGS-traps), the extend of prevention (e.g. source reduction) and the number of sterile males released for SIT and BSIT (all other parameters being kept constant at their reference value). Three periods of actions were tested: early in the year (Early) when the mosquito population is low, midway in the year (Mid) when the population is increasing, and later in the year (Late) when the population reaches its maximum. Resilience is given in number of days. Vector control actions were simulated on average meteorological dynamic and outputs were averaged over the 4 parcels studied(see “Methods”). Red diamonds indicate the results of simulations for SIT and BSIT with a reference number of released males (1000 males/ha).

Of all the vector control actions tested alone throughout their respective range of applicability (Table 1), SIT and BSIT with a weekly release rate for about 4 months in both tropical and temperate climate, were by far the most effective when used at an optimal time (Fig. 2). In the temperate climate (Fig. 2A), SIT provided effective control of the mosquito population with a reduction rate close to 1 and a resilience (*i.e.*, the time it takes to regain the natural dynamics of the mosquito population in the absence of vector control, see “Methods”) of up to 3 years when used early in the year (around March), when the wild mosquito population has a low density, *i.e.* when the released:wild males ratio is at is highest. The PP-boost delivered by BSIT provided no additional benefit, except when control started later in the year, when the mosquito population reaches its peak of abundance, thus reducing the released:wild males ratio. The efficacy of both methods has been greatly reduced in the tropical climate, where mosquito abundance remains high throughout the year although BSIT proved to be more effective than SIT, with a reduction rate of 0.77 *vs* 0.41, respectively; resilience was also doubled with the use of BSIT compared to SIT (Fig. 2B).

The other vector control methods, *i.e.*, prevention, ovitraps, BGS-traps and ADT, showed very low resilience compared to SIT and BSIT (less than one year in both climates). In the temperate climate, the reduction rate provided by these methods was also much lower than SIT ($<0.35$). In the tropical climate, a high reduction rate (0.69), 1.7 times higher than that of SIT, potentially could be achieved by using ADT.

**Figure 3:**
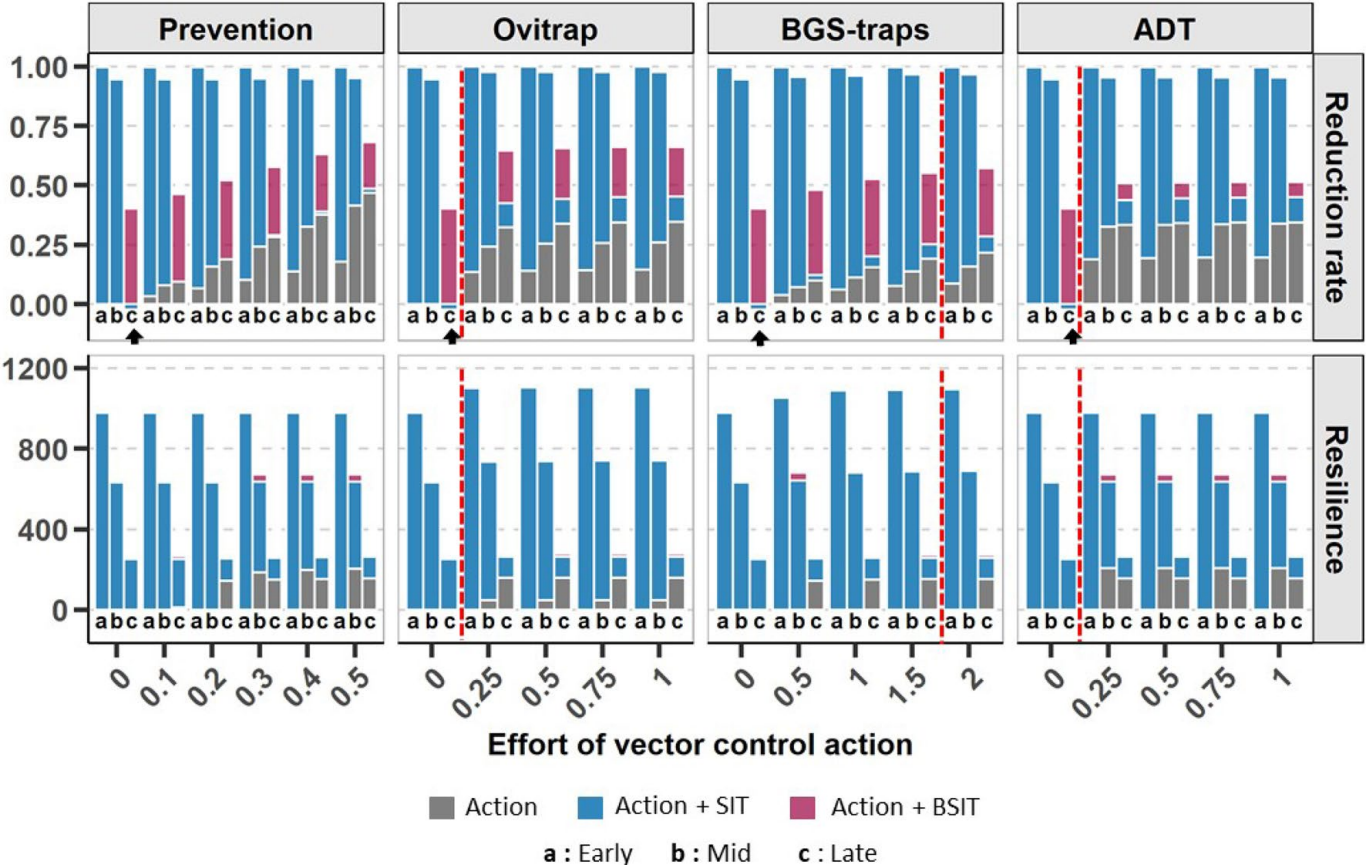
Reduction rate and resilience in the temperate climate for an increasing effort in vector control actions against *Aedes albopictus*. Vector control actions (ovitraps, BGS-traps, ADT and prevention) are represented by grey bars. The benefits added by combining them with (1) SIT (releases of 1000 males/ha) and (2) BSIT (releases of 1000 males/ha) are represented by pink and blue bars, respectively. The effort devoted to each control action is indicated, either as a rate of breeding sites destroyed for prevention, or as the number of traps/stations per house for ovitraps, BGS-traps and ADT. Three control periods were tested: early in the year (Early) when the mosquito population is low, midway in the year (Mid) when the population is increasing, and later in the year (Late) when the population reaches its maximum. The vector control actions were simulated on a mean weather dynamic and outputs were averaged among the 4 studied parcels (see “Methods”). The red dashed line indicates the number of ovitraps, BGS-traps and ADT stations required to reach the plateau of maximum effect for the action performed alone. The black arrows show the very specific case of the negative reduction rate alone caused by late releases of SIT without any other vector control action.

**Figure 4:**
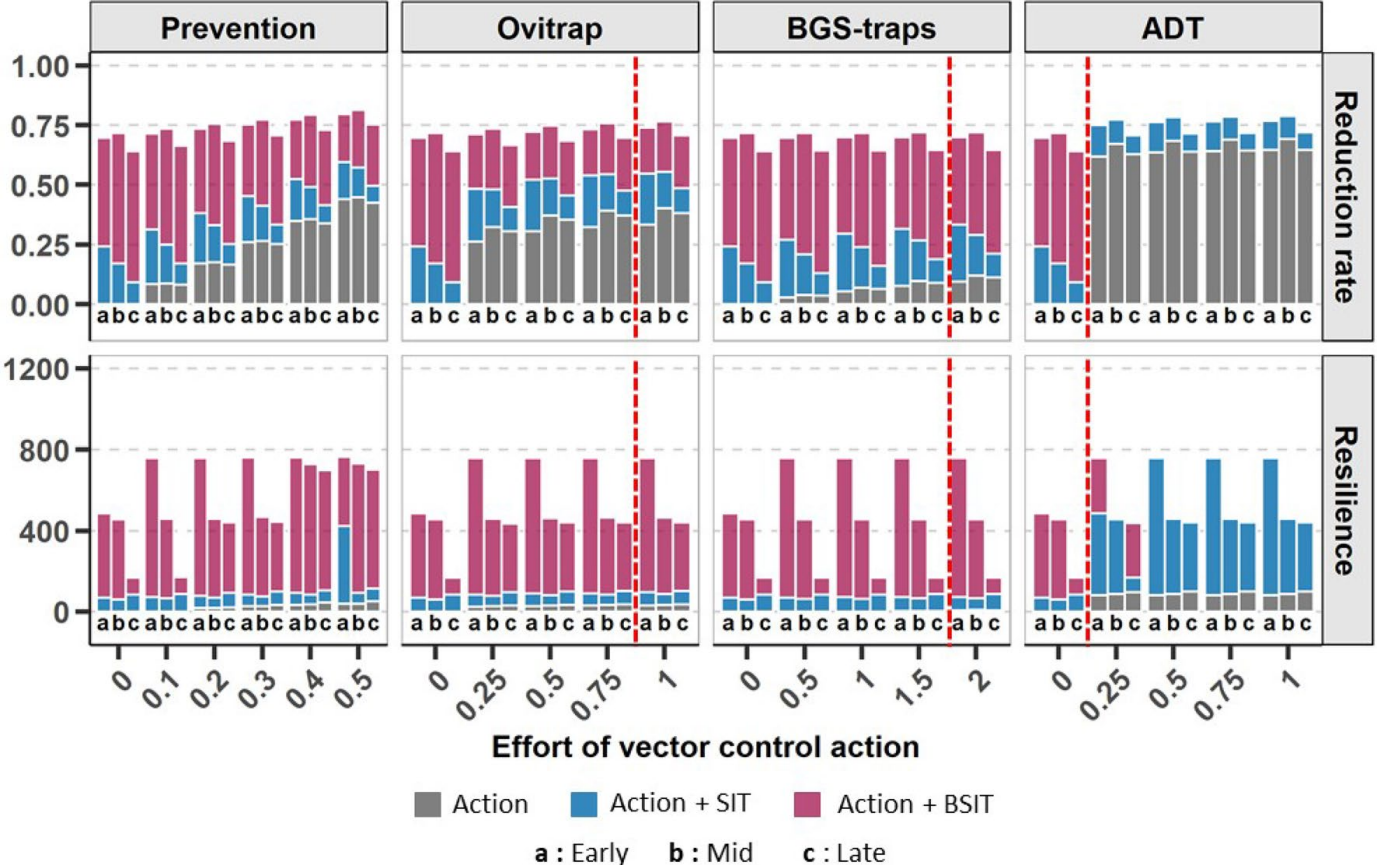
Reduction rate and resilience in the tropical climate for an increasing effort in vector control actions against *Aedes albopictus*. Vector control actions (ovitraps, BGS-traps, ADT and prevention) are represented by grey bars. The benefits added by combining them with (1) SIT (releases of 1000 males/ha) and (2) BSIT (releases of 1000 males/ha) are represented by pink and blue bars, respectively. The effort devoted to each control action is indicated, either as a rate of breeding sites destroyed for prevention, or as the number of traps/stations per house for ovitraps, BGS-traps and ADT. Three control periods were tested: early in the year (Early) when the mosquito population is low, midway in the year (Mid) when the population is increasing, or later in the year (Late) when the population reaches its maximum. The vector control actions were simulated on a mean weather dynamic and outputs were averaged among the 4 studied parcels (see “Methods”). The red dashed lines indicate the number of ovitraps, BGS-traps or ADT stations required to reach the plateau of maximum effect for the action performed alone.

Finally, for both climates, the efficiency of prevention was directly correlated to the effort put into the method, represented by the rate of breeding sites destroyed (Figs. 3, 4). In contrast, the reduction rate obtained using ADT, ovitraps and BGS-traps reached a plateau after which increasing the effort, *i.e.*, adding devices, did not improve the effect. As shown in Figs. 3 and 4, the optimal number of devices were 1 ovitrap for 4 houses in a temperate climate and 1 per house in a tropical climate, and more than 2 BGS-traps per house or 1 ADT station for 4 houses in both climates.

### While other control methods are more efficient in large mosquito populations, sterile male releases should start early in the season

As indicated above, SIT was generally more efficient (higher reduction rates and greater resilience) when it began early in the year (Fig. 2), when mosquito abundance is low (supporting results in Appendix A). For subsequent releases, the reduction rate is reduced by a maximum of 10-fold in the temperate climate and by a maximum of two-fold in the tropical climate (Fig. 2). While the effect of BSIT was similar in the temperate climate, the optimal release period for BSIT was later in the tropical climate, when the population starts to increase (Mid), favouring PP transfer between males and females; however, the longest resilience for BSIT was obtained when mosquito abundance was low, *i.e.*, early in the year.

Surprisingly, SIT can cause a temporary increase in the female population when performed during peak abundance in a temperate climate (see Appendix B). This increase is specific to releases of less than 1,100 males per hectare (Figs. 2A, 3) and is not observed in the tropical climate (Figs. 2B, 4), where the population is more stable throughout the year and does not show such a high growth rate. This undesirable effect on the population is probably due to a reduction in larval competition, since it disappears when the density-dependent terms of the model are removed (see Appendix B).

The efficiency of the other vector control actions also depends on their timing (Fig. 2). Breeding site destruction and traps/stations were more effective for intermediate to large populations in both climates (reduction rate, Fig. 2). The longest resilience was also observed for actions performed later in the year (about five months in the temperate climate), although resilience was much lower (about three weeks) in the tropical climate (resilience, Fig. 2).

### Vector control actions can be advantageously combined

By combining SIT or BSIT (releases of 1000 males/ha, see “Methods”) with other vector control methods, the observed responses were different and depended on the climate (Figs. 3, 4).

In the temperate climate, the combination of SIT with any other vector control action did not improve the reduction rate produced by SIT alone in the optimal period (*i.e.*, early treatment; Fig. 3), although resilience could be extended ($\sim 4$ months) with the use of traps at low density (3 BGS-traps or 1 ovitrap per 4 houses). When the mosquito population was high (late releases), BSIT with prevention or traps (ovitraps, BGS-traps) appeared to be the best combinations: the reduction rate could be increased by $26\%$ with the destruction of 50% of the breeding sites, or with the use of ovitraps (any effort). BSIT and ADT are redundant for breeding site contamination, so that their combination appeared unnecessary. Finally, combining actions prevented the population increase due to the late use of SIT (see above).

In the tropical climate, BSIT was more efficient than SIT alone (Fig. 4). The combination of BSIT with prevention or ovitraps could slightly increase the reduction rate (up to $14\%$ with the destruction of $50\%$ of the breeding sites and up to $7\%$ for 1 ovitrap per house), but BGS-traps did not improve it, and the combination of BSIT and ADT showed no marginal gain of performance. However, combining BSIT with prevention, ovitraps or BGS-traps early in the season could greatly improve resilience ($+9$ months) without a significant decrease in the reduction rate. Moreover, this increase in resilience was observed for a small effort on vector control actions: 10% of prevention (i.e., destruction of $10\%$ of the breeding sites), 1 ovitrap, BGS-trap or ADT station for 4 houses. Finally,simulations showed that the combination of SIT with ADT stations produced a higher reduction rate ($0.79 \pm 0.003$) than BSIT used alone (0.71) or in combination ($0.76 \pm 0.01$), with an effort of 1 station per 2 houses (Fig. 4).

## Discussion

Vector control measures are most effective and sustainable when they are fully integrated into a broader mosquito management approach^75^. Integrated mosquito management is not simply a matter of adding together different methods because while some may act synergistically, others may have antagonist effects, or may simply be redundant, wasting money and effort^75^. Moreover, the environment is a critical factor to consider when optimizing mosquito control methods^61^. Our weather-driven mechanistic model, validated on entomological data in a temperate (Appendix C) and a tropical environment^71^, thus provides the first estimates of the combined effect of different control methods against the tiger mosquito. The model is based on the release of sterile males (SIT or BSIT) and preventive mechanical destruction of breeding sites, mass trapping (ovitraps or BGS-traps) and autodissemination of biocides (ADT) under different environmental conditions.

### Mechanical control methods have similar effects against *Aedes albopictus* in temperate and tropical environments

According to the simulations, mass trapping (using ovitraps or BGS-traps) and prevention are the least effective control methods against *Ae. albopictus* populations, with broadly similar magnitudes in tropical and temperate environments. However, mass trapping and prevention are more efficient in a temperate environment when the population size is high, around mid-summer (*i.e.*, July-August). This is probably due to the fact that adult mosquito densities are reduced to zero during winter (whereas there are always adults in tropical environments); early in the season, the density of the adult population therefore is too low to capture a significant amount of females (Fig. 2). A field study nevertheless suggests that mass trapping methods show a significant population reduction only after a prior reduction in mosquito populations^76^.

However, no matter when the starting date falls, a critical element in the control of mosquito populations with traps is the involvement of local communities^76^. This is even more important for prevention, because vector control can be constrained when private gardens are difficult to access, hindering the exhaustive treatment of areas^77^.

### SIT is the most effective method to control* Aedes albopictus* in a temperate climate

In a temperate climate, SIT is much more effective at the beginning of the season, *i.e.*, just after the end of the diapause of *Ae. albopictus* eggs at the close of winter (Fig. Appendix A). As sterile males must compete with their wild competitors, starting the releases when the population is at its lowest increases their probability of mating with a female for a given release rate^61,68,78^.

However, even later in the season, but before the peak of abundance, the potential efficacy of SIT far exceeds that of other traditional vector control methods, so coupling vector control methods with SIT seems unnecessary in temperate environments when the releases start early enough (Figs. 2, 3). The seasonal reduction in density due to climatic conditions therefore suggests that a large investment in SIT would be more effective than investing in a combination of control methods^79^.

The limits of the effectiveness of SIT appear during late releases (June), *i.e.* during the peak in mosquito abundance. At that time, relatively few sterile males compete with their wild counterparts in the natural. The mating probability of sterile males is therefore too low to interfere with the ongoing natural dynamics Counter intuitively, however, and as shown in other studies^61,80,81^, the application of SIT during peak abundance could increase population sizes at the start of the control effort by reducing larval competition (Appendix B). In this worst-case scenario, the integration of another control method with SIT as well as the use of BSIT could then be a back-up solution; any method that reduces the mosquito population prior to the application of SIT would indeed increase the effectiveness of SIT^44,79^.

### SIT must be supported with other control methods against *Aedes albopictus* in a tropical environment

In contrast with temperate climate conditions, where only diapausing eggs survive the winter, a tropical climate offers favourable temperatures throughout the year and facilitates the continuous dynamics of all stages of *Ae. albopictus* populations^71^. The seasonal reduction in mosquito density is therefore too limited to allow effective population control by SIT alone, taking into account the actual feasibility for release rates ( 1000 males/ha) (Fig. 2). In this context, boosting SIT with pyriproxyfen (BSIT) and the combination of SIT with ADT have been shown to be the two most effective combined control methods. The action of pyriproxyfen lasts longer in tropical climates due to the continuous dynamics and more abundant populations of *Ae. albopictus* throughout the year. Moreover, the transmission mechanisms of pyriproxyfen and the skip-oviposition behaviour of females for both methods are more effective with slightly larger mosquito populations (*i.e.*, mid release period in tropical climate, Fig. 4 and late release period in temperate climate, Fig. 3), leading to more effective control^47,71,82^. They therefore also make it possible to delay when control actions are implemented.

Coupling BSIT with prevention or ovitraps does not significantly increase the rate of reduction, but it does double the resilience of control if implemented at an early stage. BGS-traps do not appear to have a significant effect on control, probably because they also capture sterile males, but they also do not interfere with the effectiveness of SIT or BSIT (Fig. 4).

Finally, the best combination in tropical environments seems to be SIT + ADT, with the highest reduction rates and the longest resilience time obtained from only 1 station every 4 houses, with the increased effort reaching a plateau of efficiency (Fig. 4). However, this plateau is likely to depend on variables such as the density of local populations of *Ae. albopictus* or the type of housing in the intervention area.

### Further developments: towards an integrated operational tool

The weather-driven model presented in this study accurately describes the population dynamics of *Ae. albopictus* in different environments. However, the parameters used were chosen from bibliographical and experimental knowledge, and several parameters and processes, in particular for BSIT, remain unquantified. For those cases, we chose conservative assumptions. For example, we neglected the potential direct transmission of pyriproxyfen from males to breeding sites^83^, as the number of males caught in ovitraps is low compared to females^84^. Such conservative assumptions could lead to an underestimation of the BSIT effect. Furthermore, BSIT and SIT efficiency depends on various parameters that interact with each other as the male’s mating competitiveness and, the rate, the size and the starting date of releases^71^ (Appendix 9). The applicability of each combination of parameters in the field is difficult to assess due to technical limitations or costs that are still poorly known. The scenarios presented here (Figs. 3, 4), which focus on the starting date of releases, were chosen to discuss a realistic plan of vector control actions in terms of feasibility and cost. However, the model could be easily adjusted if more precise measurements are published in the future.

Another potential limitation is that populations are modelled independently, effectively as isolated populations. As the dimensions of the parcels in Montpellier and Reunion Island (more than 5 and 4 ha respectively) are larger than the active flight distance of *Ae. albopictus* (less than 100 m^85,86^), it seems reasonable to neglect the dispersion of mosquitoes (arrival or departure of individuals). However, a recent pilot trial of transgenic male releases in Brazil showed that it is very difficult to eliminate non-isolated mosquito populations^87^. Indeed, due to their high fertility, a few *Ae. albopictus* females could have a significant impact when population numbers are low, which could significantly reduce the expected resilience^71^. The integration of limited adult migration would therefore be a crucial development to provide more robust predictions.

Despite these limitations, our model can nevertheless be easily used as an operational tool for decision-making, allowing the *in silico* experimentation of various vector control strategies. By computing the life cycle of *Ae. albopictus* in detail, the modelling framework developed is flexible in design, so that any control protocol or integrated strategy, including the sequential implementation of different methods, can be tested easily. A previous version (without any control action implemented) is in fact already routinely used by the services in charge of vector control on Reunion Island to predict *Ae. albopictus* densities over the entire island and identify priority intervention sites^88^. The current version of the model allows early planning, so that vector control stakeholders can test their own control scenarios. This model could easily be set up to run in an area where *Ae. aegypti* is the main vector since the latter shares similar traits with *Ae. albopictus*.

Our model also could be used to test additional vector control strategies. Indeed, in this study, we focused on innovative control methods which are currently in the testing phase on Reunion Island and/or in Montpellier, but other control methods exist^25,89^. These methods include the Incompatible Insect Technique (IIT) and the Release of Insects carrying a Dominant Lethal (RIDL), which are strategies based on the release of modified males inducing a reduction in the descendants^40,90^. For example, a combination of SIT + IIT made it possible to suppress *Ae. albopictus* populations from an island in China^42^. Likewise, we focused on the autodissemination of pyriproxyfen, but other biocides could be considered such as densoviruses^91^. The advantage of our mechanistic model is that it details the life cycle of *Ae. albopictus* and thus it is possible to introduce the effects of many strategies.

Furthermore, this model could help public health services as its structure allows it to be coupled with an epidemiological model. Such a combined model would allow one to study not only the impact of vector control methods^67,69,92–94^, but also the effect of vaccination^95^ or patient isolation^96^ on the basic reproduction rate ($R_0$) of vector-borne diseases, in particular for dengue. The ensuing dengue propagation modelled could then be compared to observed field data^97,98^. Thanks to its relatively simple visual displays and its versatility, our model could be used to increase community awareness and involvement. By implementing different actions and visually comparing their impacts, it could help in mobilizing the public, which could have a significant impact on the control of mosquito populations^99,100^. For example, it could help to increase the use of traps and limit the number of human breeding sites^76^, which would contribute to better management and long-term sustainability of mosquito populations^101,102^. Finally, provided that the costs of the different vector control measures are known, our model could help to study the economic aspects (cost-benefit ratio) of vector control^103^. Of note, a comprehensive study should also include all the potential benefits for society, such as, for example, the preservation of biodiversity with the implementation of an integrated strategy based no longer primarily on insecticide treatments but on a set of control measures that are equally effective but environmentally friendly^104^.

## Methods

### Modelling the effects of SIT and BSIT

To model the effects of SIT and BSIT on *Ae. albopictus* populations in a temperate climate, we adapted the model developed for Reunion, a French island with a tropical climate^71^. It is a stage-structured continuous model of differential equations composed of 11 compartments (Fig. 1; the complete model is given in Appendix D) :

*i*) Seven compartments describe the mosquito’s life cycle: eggs (*E*), larvae (*L*), pupae (*P*), emerging females ($F_{em}$), nulliparous females ($F_n$), parous females ($F_p$) and males (*M*). The only difference between the tropical and the temperate climate (apart from the parameters values) is that the *z* parameter has been added in the latter to take into account the winter season. This allows the inclusion of a diapause period during which the transition from eggs to larvae is stopped, similar to the model proposed by Tran *et al.*^74^ (supplementary information is in Appendix D).

*ii*) The last four compartments model SIT and BSIT control methods: released males, either sterile-only in the case of SIT ($M_s$), or sterile and pyriproxyfen-coated (PP-coated) in the case of BSIT ($M_{sc}$), sterile females ($F_s$) and contaminated breeding sites $B_c$ (Fig. 1). Vector control begins at $T_{start}$ and ends after $\tau$ days. During this period, $\lambda _X$ sterile males, with $X = M_{sc}$ or $M_{s}$ respectively PP-coated or not, are released every $\Delta _t$ days (pulsed releases). They die at a rate of $\mu _{M_s}$ (or $\mu _{M_{sc}}$ for PP-coated males). The probability that these sterile males, PP-coated or not, mate with emerging females ($F_{em}$) depends on their relative competitiveness $\omega$ and abundance ($M_{sc}$ and $M_s$ respectively) compared to wild males (*M*), and determines the proportion of $F_{em}$ females that become sterile females ($F_s$). Moreover, for BSIT specifically:

1. PP-coated sterile males ($M_{sc}$) transfer some PP to all females they mate with, until their coating disappears after $\kappa _F$ matings, at which time they become $M_{s}$ males;
2. PP-contaminated females disseminate the contaminant (PP) in $\kappa _{B_c}$ breeding sites while laying eggs at each gonotrophic cycle ($\gamma _{gc}$);
3. in these $\kappa _{Bc}$ PP-contaminated breeding sites, the larvae have a probability $\phi$ to survive and pupate, which affects the total pupae emergence rate;
4. PP degrades in these breeding sites, which therefore decontaminate at a rate $\nu$.

Environmental conditions have an impact on the population dynamics of *Ae. albopictus* in different parts of the model: (1) temperature has an impact on the development time of aquatic stages and the mortality of larvae (*L*), pupae (*P*) and adult females ($F_{em}$, $F_n$, $F_p$), (2) rainfall affects the number of available breeding sites and their carrying capacities ($k_L$, $k_P$), and (3) heavy rainfall has an impact on the mortality rates of aquatic stages by washing out breeding habitats. Larval and pupal competition was modelled by density-dependent functions^74^. The study area is divided into independent parcels (no mosquito dispersion or interaction between parcels) that take into account the spatial heterogeneity of the distribution of breeding sites.

Parameter estimates were based both on expert knowledge and the literature. Parameters values for SIT and BSIT are presented in Table 1; the values of the model life cycle parameters in temperate conditions are presented in Appendix E; see^71^ for the life cycle parameters values in tropical conditions. The modelled population dynamics for temperate conditions without any vector control actions have been validated on entomological data (Appendix C).

**Table 1:** Parameters values of vector control methods for tropical and temperate climate.

Parameter	Definition	Value	Range	Reference
$T_{start}$	Releases starting time	–	1 Jan.–31 Dec.	Current work
$\tau$	Release period length (days)	126	[30–180]	^41^
$\Delta _t$	Time between two releases (days)	7	[5–10]	^41^
$\lambda _{M_s}$,$\lambda _{M_{sc}}$	Number of sterile males released (ha^-11^)	1000	[600–6000]	^41^
$\omega$	Sterile male competitiveness	0.23	[0.01–0.9]	^105,106^
$\mu _{M_s}$, $\mu _{M_{sc}}$	Sterile male mortality	0.086	[0.065–0.18]	^47^
$\kappa _F$	Number of contaminating matings	1	[1–8]	Current work
$\kappa _{Bc}$	Number of contaminating ovipositions	1	[1–8]	Current work
$\nu$	Duration of larval sites contamination (day^−1^)	1/33	[1/100–1/5]	^47^
$\phi$	Proportion of larvae surviving PP exposure	0.3	[0.02–0.5]	^47,83^
$r_{prev}$	Rate of breeding sites destruction	–	[0–0.5]	Current work
$S_T$	ADT stations density (/house)	–	[0–2]	^107,108^
$O_T$	Ovitraps density (/house)	–	[0–2]	^76^
*BGS*	BGS-traps density (/house)	–	[0–2]	^31,109^
$\alpha _g$	Trap or station attraction for gravid females	6.984	–	^110^
$\alpha _{hs}$	BGS-trap attraction for host -seeking females	0.52	–	^111^
$\alpha _{M_{all}}$	BGS-trap attraction for males	0.24	–	^111^
$\varepsilon$	Proportion of males landing on feeding sources	0.0244	–	^111^

### Modelling the effects of the other control methods

We then extended the model to simulate the effect of several alternative control methods, based on mechanical prevention, ovitraps, adult traps and larvicide autodissemination stations (ADT). For these methods, we assumed that they were applied for a specific period of time at a constant intensity and uniformly throughout the area. After this period, the system returned to its initial state. They were computed independently or in combination with SIT or BSIT (the complete model is given in Appendix D). Parameter estimations and their respective ranges were based on both expert knowledge and data from the literature (Table 1) in order to obtain practical levels of inputs.

#### Prevention

Prevention, *i.e.*, the mechanical destruction of potential breeding sites, was mathematically implemented in the form of a reduction of the number of available breeding sites, expressed as a percentage of the initial values ($B_{tot}(1-r_{prev})$), and thus of the carrying capacities $\left( k_x(1-r_{prev}) \text { with } x \in \{L, P\}\right)$ for larvae and pupae, respectively.

#### BGS-traps

Commonly used BGS-traps capture both females ($F_{hs}$) and males ($M_{all}$). Mass trapping control was implemented in the form of an additional mortality rate due to capture, $c_{x,BGS}, \text { with } x \in \{F_{hs}; M_{all}\}$. We assumed that any adult mosquito entering the trap would die:

1. Females ($F_{hs}$) are caught when seeking a host, *i.e.*, parous or nulliparous females; their capture rate was thus $\gamma _{gc}c_{F{hs},BGS}$ per day. The probability of capture of females ($c_{F{hs},BGS}$) was estimated by the relative availability of traps, weighted by their attractiveness for females ($\alpha _{F_{hs}}$), compared to other blood-feeding sources, *i.e.*, the number of humans living in the area $N_{tot}$ (Eq. 1).
2. Males, wild or sterile ($M_{all}=M+M_s+M_{sc}$), are captured while searching for a mate; their daily capture rate depends on the probability that a male will land on the female’s blood-feeding source ($\epsilon$) and that this feeding source is in fact a trap ($c_{M_{all},BGS}$), and is therefore expressed by $\epsilon c_{M_{all},BGS}$. We conservatively neglected the fact that males could also be trapped when flying near the trap. The probability of males being caught was therefore estimated by the relative availability of traps, weighted by their attractiveness to males ($\alpha _{M_{all}}$), compared to the number of females on other potential blood-feeding sources, again $N_{tot}$ (Eq. 1).

1 $$\begin{aligned} c_{x,BGS} = \frac{\alpha _{x}BGS}{\alpha _{x}BGS+N_{tot}} \quad \text {with} \quad x \in \{F_{hs}; M_{all}\} \end{aligned}$$

#### Ovitraps

Gravid females are attracted to ovitraps when they are looking for an ovipositing site. We assumed that only females were caught by the ovitraps (no males) and that any female entering the trap would die with her offspring. This was implemented by adding a specific mortality parameter ($c_{F_g, O_T}$), equal to the probability of being caught, for nulliparous ($F_n$) and parous ($F_p$) females. The probability of females being captured by ovitraps is therefore the ovitraps density ($O_T$) weighted by the relative attractiveness of ovitraps ($\alpha _{F_g, O_T}$) among all the available breeding sites, *i.e.*, breeding sites ($B_{tot}$) or ovitraps (Eq. 2).

2 $$\begin{aligned} c_{F_g,O_T} = \frac{\alpha _{F_g} O_T}{B_{tot}+\alpha _{F_g} O_T} \end{aligned}$$

As a female can be captured only once per gonotrophic cycle, the ovitrap capture rate is thus $\gamma _{gc}c_{F_g, O_T}$.

#### Autodissemination (ADT)

Similarly, gravid females may be attracted to ADT stations when looking for an ovipositing site. The main difference is that females entering ADT stations do not die, instead they are coated with PP and contaminate the breeding sites which they visit later. Contamination of gravid females ($c_{F_g,S_T}$) was described by their probability of entering ADT stations instead of a breeding site: we used the same approach as for ovitraps (Eq. 2), replacing the density of ovitraps $O_T$ by the density of ADT stations $S_T$. We assumed similar attractiveness for ovitraps and ADT stations, and again that no males were caught.

We modelled the contamination of the breeding sites visited later as for BSIT (see above): at each gonotrophic cycle ($\gamma _{gc}$), contaminated females ($c_{F_g,S_T}(F_n+F_p+F_s)$) laying in an uncontaminated laying site (in proportion $\frac{B_{tot}-B_c}{B_{tot}}$) transfer part of their PP-coating to it ($\kappa _{Bc}$). The number of newly-contaminated breeding sites thanks to ADT stations is therefore $c_{F_g,S_T}(F_n+F_p+F_s)\kappa _{Bc}\gamma _{gc}\left( \frac{B_{tot}-B_c}{B_{tot}}\right)$.

### Initial conditions and simulations

The model was implemented in R (http://www.rproject.org/). The numerical solutions were estimated using the implicit Runge–Kutta method from the DeSolve package.

At $t_0$, the population in each parcel consisted of $10^6$ eggs (stage *E*).

To assess the effect of vector control actions in a tropical climate, simulations using tropical parameter values were performed on four parcels from the North, South, East and West of Reunion Island. Each parcel was associated with the nearest meteorological station to drive the population dynamics. Due to inter-annual weather variations, we worked with the average daily temperature and rainfall recorded from 2012 to 2016 on the island.

Key parameters that affect the efficiency of SIT and BSIT in a temperate climate were studied by performing a sensitivity analysis (Appendix F) based on a range of realistic settings for SIT and BSIT (Table 1). The model was also used to assess the effect of vector control actions in a temperate climate. Five years of weather records (2014-2018), daily temperatures and rainfalls, provided by the French meteorological organization, Météo France, were used as inputs. The model was run for *i)* parcels corresponding to five residential areas for which entomological data were available to validate the model (Appendix C), and *ii)* four parcels with the same characteristics (size and carrying capacities) as the parcels on Reunion Island to compare the results in temperate and tropical climates.

### Numerical analysis of vector control efficiency

#### Model outputs

We focused our analyses on two outputs from the model of Haramboure *et al.*^71^:

- The **reduction rate** was computed by dividing the abundance of fertilized females during the vector control period by the abundance they would reach at the same time in an untreated population, minus 1 (reduction);
- The **resilience**, *i.e.*, the number of days after the end of the control required for the population abundance to reach a similar level (less than 10% difference) to that of a population without vector control. Resilience was computed on eggs and adult females.

These two outputs were averaged over the parcels studied to give an overall value for each scenario of vector control action.

#### Effectiveness of vector control methods

The effects of ovitraps, ADT stations, BGS-traps and mechanical prevention have been assessed in different scenarios, alone and in combination with either SIT or BSIT. In combination, it was assumed that the two vector control methods were applied simultaneously, during the same time period. To provide realistic scenarios^41^ and to reveal potential interactions between the methods, the number of males released, the release rate and the release period were set at their reference value (Table 1) in SIT and BSIT. The resilience and reduction rate were compared to determine whether SIT conferred a net benefit over the other control method alone, and whether BSIT could increase this benefit. The outputs of these two models were computed for different levels of effort in prevention ($r_{prev}$), and for different densities of trapping devices (*BGS*, $O_T$) or ADT stations ($S_T$) (Table 1).

Finally, three periods of vector control were defined according to the abundance of mosquitoes: (1) the end of the winter, when the population is lowest; (2) the beginning of the summer, when the population begins to increase; and (3) the end of the summer, when the population has reached its peak (Table 2). They were tested in independent scenarios, respectively named “Early release”, “Mid release” and “Late release”. The date of releases for the “Early release” scenario was defined based on the basis of the best release date for SIT and BSIT, computed by an optimization process (see Appendix A).

**Table 2:** Typical starting date for vector control defined when the mosquito population a) is at its lowest (Early release), b) begins to increase (Mid release) and c) has reached its maximum (Late release).

	Tropical climate				Temperate climate
	North	East	South	West	All
(a) Early	6 Aug	5 Aug	4 Sep	23 Oct	24 Mar
(b) Mid	8 Dec	1 Nov	9 Dec	24 Dec	20 May
(c) Late	21 Jan	29 Jan	13 Feb	5 Feb	17 Jun

Given the wide climatic variations within Reunion Island, the three vector control periods were specific to each zone, North, East, West and South in tropical climates, whereas in temperate climate, a single configuration for each period was applied on all parcels (Table 2).

## Supplementary Information

Supplementary Information
