# Supplementary material for: Comparing sterile male releases and other methods for integrated control of the tiger mosquito in temperate and tropical climates
Source: Sci Rep. 2021 Apr 1;11:7354. doi: 10.1038/s41598-021-86798-8 (PMC8016901; doi:10.1038/s41598-021-86798-8)
Supplement: Supplementary file 1 — Supplementary Information [file 41598_2021_86798_MOESM1_ESM.pdf]

A Optimization of the sterile males releases date

To identify the best period for the sterile males releases, we optimized the starting date of releases similarly to Haramboure *et al.*[1]. The effectiveness of the SIT and BSIT was quantified by gathering 5 model outputs into a single value (1): the mean reduction rate over the releases (no units), the resilience (years), the sterility rate (no units), the emergence reduction rate (no units) and the increase rate of female population (no units). These outputs were computed relatively to the control model (without any vector control actions). Since the increase in female population during releases is an undesirable effect, the female increase rate was substracted to lower down effectiveness estimator. The release starting day (T_{start}) was optimized to find the overall maximal effectiveness value. Both global and local optimizations were performed using the algorithm from `nloptr R` package (https://cran.r-project.org/web/packages/nloptr/index.html).

$$\begin{aligned} Effectivness &= f(T_{start}) \\ &= \sum_y \sum_p Reduction\ rate_{yp} + Sterility\ rate_{yp} + Resilience_{yp} \\ &\quad + Emergence\ reduction_{yp} - Increase\ rate_{yp}, \end{aligned} \tag{1}$$

$$\text{with} \begin{cases} p & \in \text{Parcels}, \\ y & \in [2015-2017]. \end{cases}$$

Optimization of SIT and boosted SIT consisted of maximizing their respective effectiveness. Because of inter-annual weather variations and field heterogeneity, these effectiveness were computed in three different years (2015, 2016 and 2017) and on the four Reunion Island-like parcels linked to the Montpellier meteorological stations (see Methods). It thus takes into account weather variability and field heterogeneity in both SIT and boosted SIT efficiency.

We defined the best release period as the time-frame of vector control actions that allows to reach at least 95% of the maximal potential effectiveness.

The best starting period was found to be late March in the temperate climate. A 3.5-months interval extending from February to mid-May allows reaching at least 95% of the maximum SIT and BSIT potential (Figure 1(A)). The 99% interval, mainly driven by resilience augmentation, is much shorter and lasts 40 days for both SIT and BSIT. It takes place within the firsts months of the favorable period, when the adult population is very low (Figure 1 (B)).

During control, and similarly in SIT and BSIT, the female population is heavily lowered during summer, approaching extinction (Figure 1 (B)). A few individuals nevertheless survive and breed, leading to the population recovery the year after.

B Undesirable increase in female abundance

When started late in the mosquito season, SIT with late releases of less than 1100 males per hectare, has been found to cause a temporary increase in female abundance (see Results), which is not a desirable effect of control. This undesirable increase of female abundance has been reported in several studies[2–4]. We further investigated the causes by running two sets of three simulations for SIT, BSIT and a control, one with larval competition and the other without (*i.e.*, removing the competition term $1+\frac{P}{k_P(\mathbf{1-a\ r_{prev}})}$, eq. (2)). To allow comparison while avoiding the burst in population induced by the removal of larval competition, the total numbers of adult males and females were caped at 200 individuals.

An increase in female abundance was observed for SIT, which disappeared with the removal of larval competition from the model (Figure 2), suggesting that the sterile males releases induce a reduced larval competition, leading temporarily to more emergence. This increase is not observed with BSIT, either with or without larval competition, probably because the pp-contamination of breeding sites heavily lower down the larval population and overcome the effect of any competition process.

C Model validation in the temperate climate

The simulated population dynamic without any sterile male releases (eq. (2) : $\lambda_{M_s} = \lambda_{M_{sc}} = 0$ and $a = 0$) has been validated on entomological data recorded by the "Entente Interdépartementale de Démoustication (EID) Méditerranée", the public agency in charge of the mosquito control in the southeast France.

Ovitraps were placed in 5 residential areas (Table 1) located in Montpellier (Vert-bois, Pompigane Sud and Aiguelongue) or in the adjacent city of Castelnau-Le-Lez (Volhe and Chevalerie) (Figure 3). Studied areas were described as discontinuous medium to dense urban fabrics by the reference CORINE, an European reference describing the land-cover structure based on

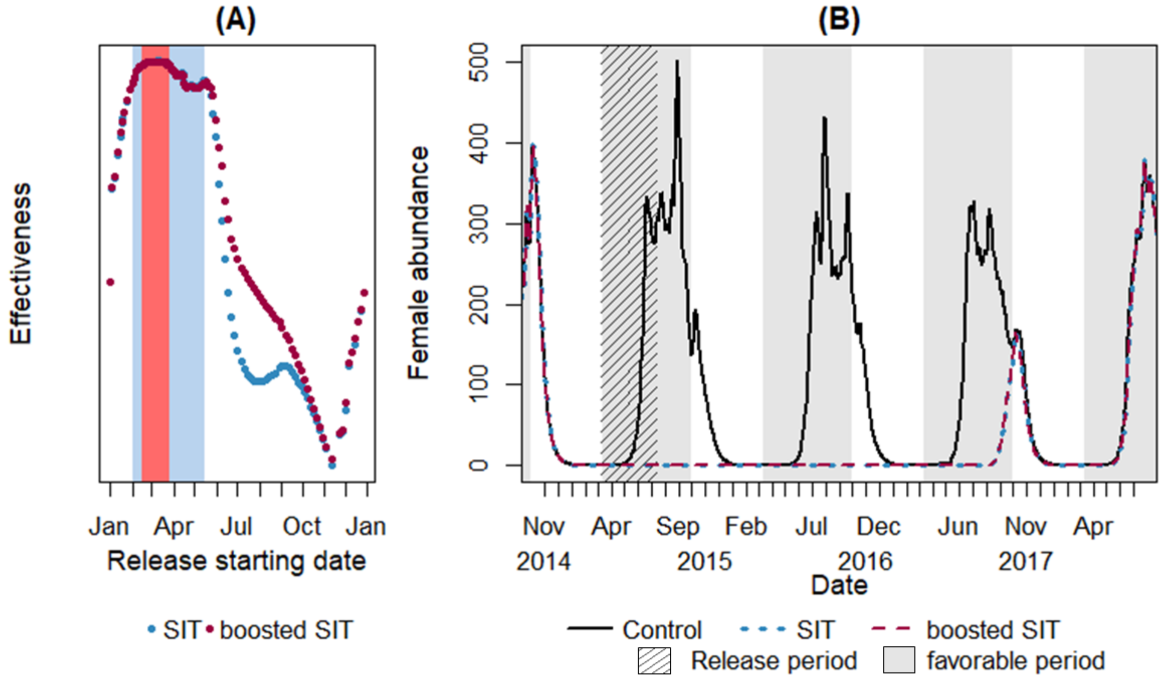

Figure 1. Best release starting date in the temperate climate for a maximum SIT and BSIT effect. (A) Effectiveness at different starting dates for SIT (blue) and BSIT (red). Periods reaching at least 95% and 99% of the maximum effect are respectively filled in blue and red. (B) SIT and BSIT effect on female dynamics over time. The release starts at its optimal day (March, the 24th) in year 2015. The scenario consists of 18 releases of 1,000 males/ha during a period of 4 months. Grey background represents the mosquitoes favorable period, *i.e.*,. when no diapause occurs.

satellite imagery[5]. Ovitraps were mostly placed in sites shaded by vegetation. They consisted of 3L plastic black buckets filled with 2L of tap water, in which a floating polystyrene square (5 x 5cm) acted as an ovipositing support. Biolarvicide *Bacillus thuringiensis israelensis* was added into the bucket to prevent the production of mosquitoes from the trap.

The number of trapped eggs were monitored weekly between April and November, in 2015, 2016 and 2017 (Table 1). The trapping network in each parcel was composed of 8 to 25 ovitraps. We considered that a reasonable-enough trapping effort was performed to get an accurate description of the local *Ae. albopictus* population dynamics. Hatched and unhatched eggs were removed from the trap each week for identification and count at the laboratory.

Table 1. Entomological record of *Ae. albopictus* in the Montpellier area

Location	Year	Beginning	End	Number of traps	Surface of trapping area (ha)	Maximum mean number of eggs per trap per capture session
Vert-Bois	2015	30 Avr	18 Nov	25	9.739	434.36
Vert-Bois	2016	12 Mai	24 Nov	11	9.739	395.7
Vert-Bois	2017	26 Avr	19 Oct	19	9.739	294.2
Chevalerie	2016	12 Mai	24 Nov	10	5.876	377.2
Chevalerie	2017	26 Avr	19 Oct	8	5.876	259.25
South Pompignane	2016	12 Mai	24 Nov	10	12.313	334.9
Volhe	2016	12 Mai	24 Nov	10	9.954	483
Volhe	2017	26 Avr	19 Oct	11	9.954	228.36
Aiguelongue	2017	26 Avr	5 Oct	20	5.026	200.4

These observed data were then compared to the predictions of the model using a Spearman correlation test. The predicted numbers of new eggs produced per week were independently computed as $(\gamma_{gc}(\beta_n F_n + \beta_p F_p))$. Model simulations were highly consistent with the dynamics recorded in the field, with a significant correlation of 61% ($p_{value} \simeq 10^{-27}$).

However, the model tends to underestimate the number of new eggs produced at the beginning of the favorable period (April). An early egg peak is observed in 2017 in all studied areas (Figure 4). This burst is not captured by our simulations and could

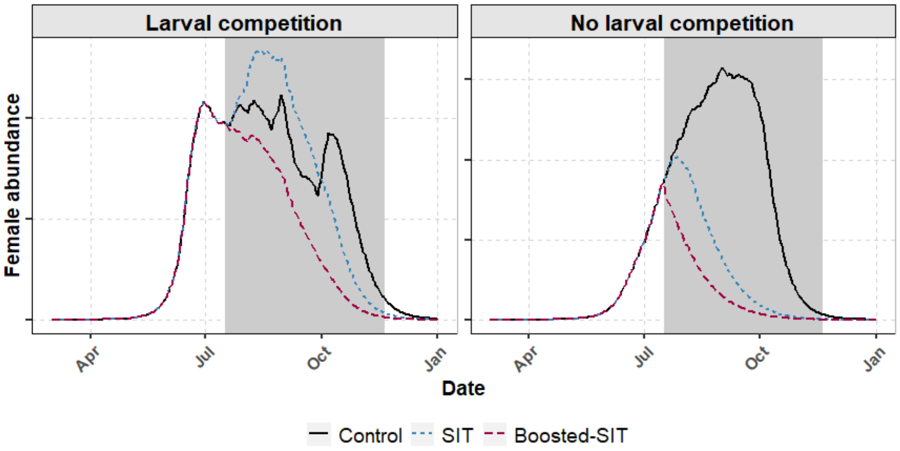

Figure 2. Population dynamics during late implementation of SIT or BSIT, with or without pupal competition. The release period is filled in grey. We bounded the adult females abundance to avoid exponential growth of population when the density-dependent (*i.e.*, the competition) process are removed.

not be explained by temperature or rainfall fluctuations. It is probably responsible for the lower correlation (52%, $p_{value} \simeq 0.01$) observed in the Aiguelongue area where the few records (24) provided were all performed in 2017. Eggs measured in Chevalerie, Volhe and Vert-bois areas, were followed for more than one year and show higher correlation coefficients: 0.75 ($p_{value} \simeq 10^{-11}$), 0.69 ($p_{value} \simeq 10^{-9}$) and 0.65 ($p_{value} \simeq 10^{-11}$), respectively. The best fit to the entomological data was observed in the South Pompignane neighborhood with a significant correlation of 86% ($p_{value} \simeq 10^{-9}$); this area is the only one where no records were performed in year 2017.

D Full model, modified from Haramboure *et al.* [1]

The original model was developed for Reunion Island (southwest Indian Ocean, tropical climate) by Haramboure *et al.*[1]. The present model is an adaptation that allows for the temperate climate, using Montpellier (southern France, Mediterranean

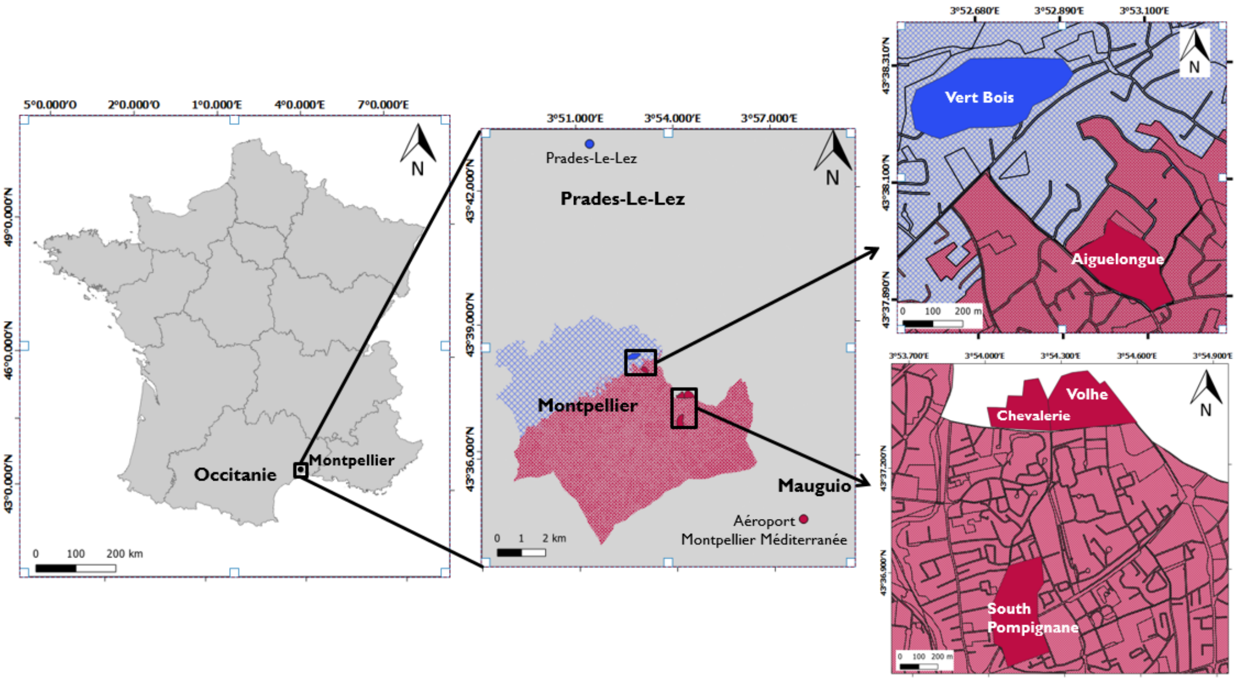

Figure 3. Study area. Colored points represent the two meteorological stations (blue for Prades-Le-Lez and red for Montpellier's airport) of the area. The same color code was used to link each parcel with its closest meteorological station. Entomological data were provided for 5 parcels : Vert-Bois, Aiguelongue, South Pompignade, Chevalerie and Volhe (filled parcels). The maps were produced using `QGIS` 3.10 (`http://www.qgis.org`). The shapefile of the regions of France is taken from the open street map (`https://www.data.gouv.fr/fr/datasets/contours-des-regions-francaises-sur-openstreetmap/`).

temperate climate, *i.e.*, hot and dry summers *vs.* mild and humid winters) as a reference:

$$
\begin{cases}
\dfrac{dE}{dt} = \gamma_{gc}(\beta_n F_n + \beta_p F_p) - (\mu_E + z f_E)E, \\
\dfrac{dL}{dt} = z f_E E - \left(m_L\left(1 + \dfrac{L}{k_L(\mathbf{1} - \mathbf{a}\,\mathbf{r_{prev}})}\right) + f_L\right)L, \\
\dfrac{dP}{dt} = f_L L - (m_P + f_P)P, \\
\dfrac{dF_{em}}{dt} = \sigma f_P P exp\left(-\mu_{F_{em}}\left(1 + \dfrac{P}{k_P(\mathbf{1} - \mathbf{a}\,\mathbf{r_{prev}})}\right)\right)\left(1 - \dfrac{B_c}{B_{tot}(\mathbf{1} - \mathbf{ar_{prev}})}(1-\phi)\right) \\
\qquad\qquad - (m_F + \gamma_{F_{em}})F_{em}, \\
\dfrac{dF_n}{dt} = \gamma_{F_{em}} F_{em}\left(1 - \dfrac{\omega(M_{sc}+M_s)}{\omega(M_{sc}+M_s)+M}\right) - \left(m_F + \mu_r + \boldsymbol{\gamma_{gc}}\left(\mathbf{1} + \mathbf{a}\left(\mathbf{c_{F_g,O_T}} + \mathbf{c_{F_{hs},BGS}}\right)\right)\right)F_n, \\
\dfrac{dF_p}{dt} = \gamma_{gc} F_n - \left(m_F + \mu_r + \mathbf{a}\left(\mathbf{c_{F_g,O_T}} + \mathbf{c_{F_{hs},BGS}}\right)\boldsymbol{\gamma_{gc}}\right)F_p, \\
\dfrac{dF_s}{dt} = \gamma_{F_{em}} F_{em} \dfrac{\omega(M_{sc}+M_s)}{\omega(M_{sc}+M_s)+M} - \left(m_F + \mu_r + \boldsymbol{\gamma_{gc}}\mathbf{a}\left(\mathbf{c_{F_g,O_T}} + \mathbf{c_{F_{hs},BGS}}\right)\right)F_s, \\
\dfrac{dM}{dt} = (1-\sigma) f_P P exp\left(-\mu_{F_{em}}\left(1 + \dfrac{P}{k_P(\mathbf{1} - \mathbf{a}\,\mathbf{r_{prev}})}\right)\right)\left(1 - \dfrac{B_c}{B_{tot}(\mathbf{1} - \mathbf{ar_{prev}})}(1-\phi)\right) \\
\qquad\qquad - (\mu_M + \mathbf{a}\boldsymbol{\varepsilon}\mathbf{c_{M_{all},BGS}})M, \\
\dfrac{dM_s}{dt} = \dfrac{F_{tot}}{\omega(M_{sc}+M_s)+M}\dfrac{\omega M_{sc}}{\kappa_F} - (\mu_{M_s} + \mathbf{a}\boldsymbol{\varepsilon}\mathbf{c_{M_{all},BGS}})M_s \\
\dfrac{dM_{sc}}{dt} = -\dfrac{F_{tot}}{\omega(M_{sc}+M_s)+M}\dfrac{\omega M_{sc}}{\kappa_F} - (\mu_{M_s} + \mathbf{a}\boldsymbol{\varepsilon}\mathbf{c_{M_{all},BGS}})M_{sc}, \\
\dfrac{dB_c}{dt} = \left(\dfrac{\omega M_{sc}}{\omega(M_{sc}+M_s)+M} + \mathbf{ac_{F_g,S_T}}\right)(F_n + F_p + F_s)\kappa_{Bc}\gamma_{gc}\left(1 - \dfrac{B_c}{B_{tot}(\mathbf{1} - \mathbf{ar_{prev}})}\right) - \nu B_c, \\
M_x(T_{start} + n\tau^+) = M_x(T_{start} + n\Delta_t) + \lambda_{M_x},\ n = 1,2,3,\ldots; \quad n\Delta_t \le \tau; \quad x \in \{sc, s\}, \\
[illegible]
\end{cases}
\tag{2}
$$

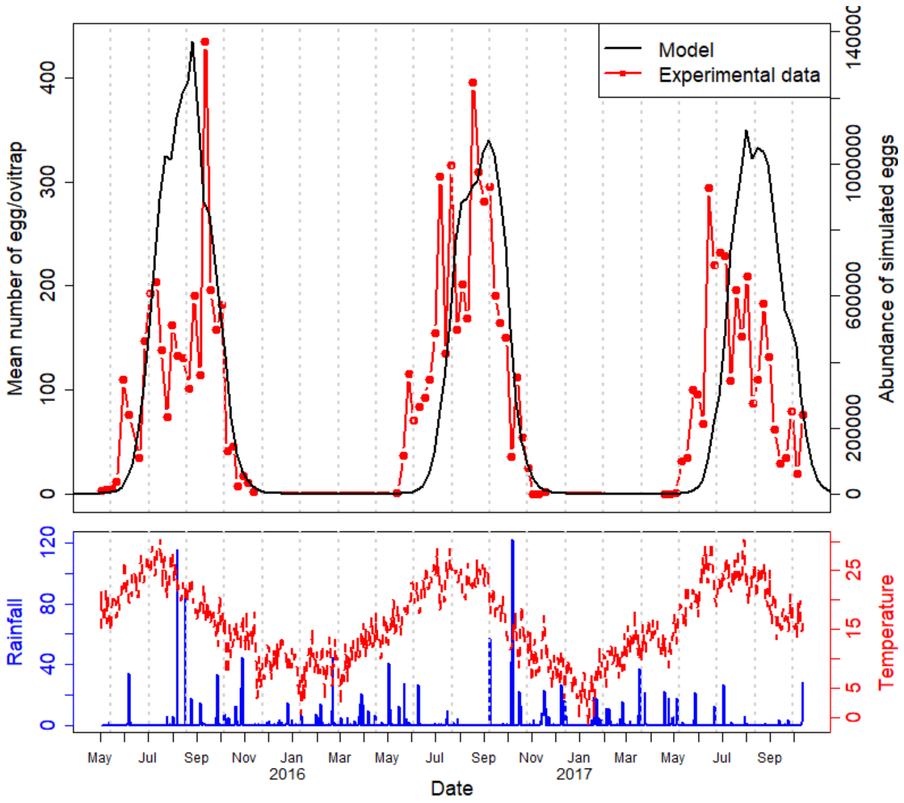

Figure 4. Model *vs.* observed data in the Vert-bois area. (Top panel) Comparison of the numbers of new eggs sampled each week in the entomological collection (red) and predicted by the model (black). (Bottom panel) Temperature and rainfall records from Prades-Le-Lez meteorological station.

$$\text{with } z = \begin{cases} 0 & \text{if } d_{start} \le t \le d_{end} \text{ (diapause)} \\ 1 & \text{otherwise} \end{cases} \qquad \text{and} \qquad \mathbf{a} = \begin{cases} \mathbf{1} & \textbf{during vector control} \\ \mathbf{0} & \textbf{otherwise} \end{cases}$$

Constant parameters are in Greek letters. They include mortality rates (μ_X being the mortality rate of compartment X, 11 compartments in total, see text), and the transition rates from emerging to nulliparous females ($\gamma_{F_{em}}$), and from nulliparous to parous females (γ_{gc}). γ_{gc} is then multiplied by the number of eggs laid per ovipositing nulliparous (β_n) and parous (β_p) females to compute the egg-laying rate. An additional mortality rate is added for female compartments, due to their host-seeking behaviour (μ_r). σ is the sex ratio at emergence. Each male being able to fertilize several females, we assumed that the mating probability for females was 1, *i.e.*, independent of the male density.

Latin letters are weather-dependent functions that act on the mortality rates (m_x), the transition rates (f_x) and the environment-defined carrying capacities (k_x) for the aquatics stages. Carrying capacities drive the density-dependent mortality at larval stage $\left(m_L\left(1+\frac{L}{k_L}\right)\right)$ and the pupae density-dependent success for adult emergence $\left(exp\left(-\mu_{F_{em}}\left(1+\frac{P}{kp}\right)\right)\right)$. Temperature impacts m_x and f_x, whereas k_x is rainfall-dependent.

During diapause (d_{start},d_{end}), observed only in the temperate climate, the transition between the eggs and larvae compartments is stopped by the z parameter that represents the egg dormancy process[6].

When SIT, or BSIT, starts (T_{start}), a quantity of sterile males (λ_X, with $X = M_s$ or M_{sc}, resp.), is periodically released into the population (Δ_t) during a given period of time τ. The sterile males have a probability to mate with an emerging females (F_{em}) depending of their relative availability compared to the overall number of males, *i.e.*, their proportion adjusted by the competition parameter ω: $p = \frac{\omega X}{\omega X + M}$. No eggs produced by sterile females will hatch. Emerging females that encounter a sterile male thus become sterile (F_s), while the other become nulliparous (F_n, probability $1-p$).

Moreover, for BSIT the released sterile males (M_{sc}) are also coated with pyriproxyfen (PP). Males attempt to mate with any females of the population ($F_{tot} = F_{em} + F_n + F_p + F_s$), even if already fertilized. Each encounter of a contaminated male with a female makes it loose a part of its PP coating until total decontamination after κ_F matings : $\left(\frac{\omega M_{sc}}{\omega(M_{sc}+M_s)+M}\frac{F_{tot}}{\kappa_F}\right)$. The eventually

decontaminated males nevertheless keep their ability to sterilize females (M_s). While laying eggs at each gonotrophic cycle (γ_{gc}), PP-contaminated females transfer the contaminant to the breeding sites $\left(\frac{\omega M_{sc}}{\omega(M_{sc}+M_s)+M}(F_n+F_p+F_s)\gamma_{gc}\right)$, but only to the non-contaminated fraction $\left(1-\frac{B_c}{B_{tot}}\right)$, with B_c the number of contaminated sites among all breeding sites (B_{tot}). Females loose part of the contaminant at each oviposition, until total decontamination after κ_{Bc} gonotrophic cycles. Pupae emergence rate is affected by the probability of the pupae to 1) grow in a contaminated breeding site and 2) to survive PP with a probability ϕ $\left(1-\frac{B_c}{B_{tot}}(1-\phi)\right)$. Finally, breeding site become decontaminated at a rate ν.

Bold sections of the equation (eq. (2)) model the vector control methods that are not based on sterile males releases : ADT (O_T, S_T), ovitraps (O_T), BGS-traps (BGS) and prevention (r_{prev}) through breeding site destruction.

E Model parameters

Parameters and weather-dependent function have been adjusted according to expert knowledge and literature (Table 2 and 3).

The density of breeding sites B_{tot} was estimated from the urbanization level in each delimited subarea, as the mean number of breeding sites per household ($10-20$) multiplied by the number of households per hectare ($25-30$) and the surface of the parcel. All were estimated from field observations, as well as the distinction between permanent and rainfall-dependent breeding sites.

Table 2. Parameters values in the temperate climate

Parameter	Definition	Value	Reference
σ	Sexe ratio at emergence	0.5	[7]
β_n	Eggs laid by ovipositing nulliparous female (per female)	95	[8]
β_p	Eggs laid by ovipositing parous female (per female)	75	[8]
γ_{Fem}	Transition rate from emerging female to nulliparous female (/day)	0.4	[6]
γ_{gc}	Length of the gonotrophic cycle (/day)	0.2	[6]
μ_E	Egg mortality rate (/day)	0.05	(Lacour, unpublished)
μ_L	Minimum larva mortality rate (/day)	0.02	(Lacour, unpublished)
μ_P	Minimum pupa mortality rate (/day)	0.02	(Lacour, unpublished)
μ_{Fem}	Mortality rate during adult emergence (/day)	0.1	(Lacour, unpublished)
μ_r	Female mortality rate related to seeking behaviour (/day)	0.058	current work
μ_M	Male mortality rate (/day)	0.0735	[7]
μ_F	Female mortality rate (/day)	0.02	[7]
$k_{L_{fix}}$	Standard rainfall-independent environment carrying capacity for larvae	$[0-55358]$	Field estimation *
$k_{L_{var}}$	Standard rainfall-dependent environment carrying capacity for larvae	$[0-40104]$	Field estimation *
$k_{P_{fix}}$	Standard rainfall-independent environment carrying capacity for pupae	$[0-55358]$	Field estimation *
$k_{P_{var}}$	Standard rainfall-dependent environment carrying capacity for pupae	$[0-40104]$	Field estimation *
T_E	Minimal temperature needed for egg development (°C)	10	[7]
TDD_E	Total number of degree-day necessary for egg development (°C)	110	(Lacour, unpublished)
d_{start}	Beginning of the diapause period	30 Sept	[9]
d_{end}	End of the diapause period	10 Mar	[9]

* Standard environmental carrying capacities were extrapolated from field estimations for each delimited sub-area[10].

F Sensitivity analysis in temperate climate

A sensitivity analysis (SA) was performed on the model outputs to identify key parameters of the SIT and BSIT in temperate climate. Four model outputs were studied : the reduction rate, the resilience, the reduction rate of BSIT compared SIT and the undesirable female augmentation.

SIT parameters (λ_{M_s}, ω, μ_{M_s}), BSIT parameters ($\lambda_{M_{sc}}$, ν, κ_{Bc}, κ_F, $\mu_{M_{sc}}$) and parameters describing the release period (T_{start}, τ, Δ_t) were varied one by one through 100 trajectories to complete a Morris Sensitivity analysis[11] with the `sensitivity R` package. The grid of parameters space was discretized by 10 levels and the walking step was defined as $\frac{10}{2}$, following Morris's recommendations.

Each parameter space (Table 1) was determined from literature or expert knowledge, except for T_{start}, which was just estimated.

Table 3. Functions adjusted for temperate conditions[6]. R is the rainfall and T, the temperature at time t.

Function	Definition	Expression
f_E	Transition function from egg to larvae	$(T(t) - T_E)/TDD_E$
f_L	Transition function from larvae to pupae	$-0007T^2 + 0.0392T - 0.3911$
f_P	Transition function from pupae to emerging adult	$0.0008T^2 - 0.0051T + 0.0319$
m_L	Larva mortality	$\mu_L + 0.0007exp(0.1838(T-10))$
m_P	Pupa mortality	$\mu_P + 0.0003exp(0.2228(T-10))$
m_F	Female mortality	$\mu_F + 0.0003exp(0.1745(T-10))$
R_{norm}	Normalized weekly rainfall amount	$\left(\sum_{i=t-14}^{t} R(i)\right)/100$
k_L	Environmental carrying capacity for larvae	$k_{L_{fix}} + min\left(k_{L_{var}} R_{norm}(t), k_{L_{var}}\right)$
k_P	Environmental carrying capacity for pupae	$k_{P_{fix}} + min\left(k_{P_{var}} R_{norm}(t), k_{P_{var}}\right)$

As previously found for the tropical climate[1], the main parameters influencing the mosquito population dynamics are essentially the same between SIT and BSIT (Figure 5). The elementary effect variance increases for most of the parameters according to the absolute value of the elementary effect. Most parameters have few or no influence on the output values.

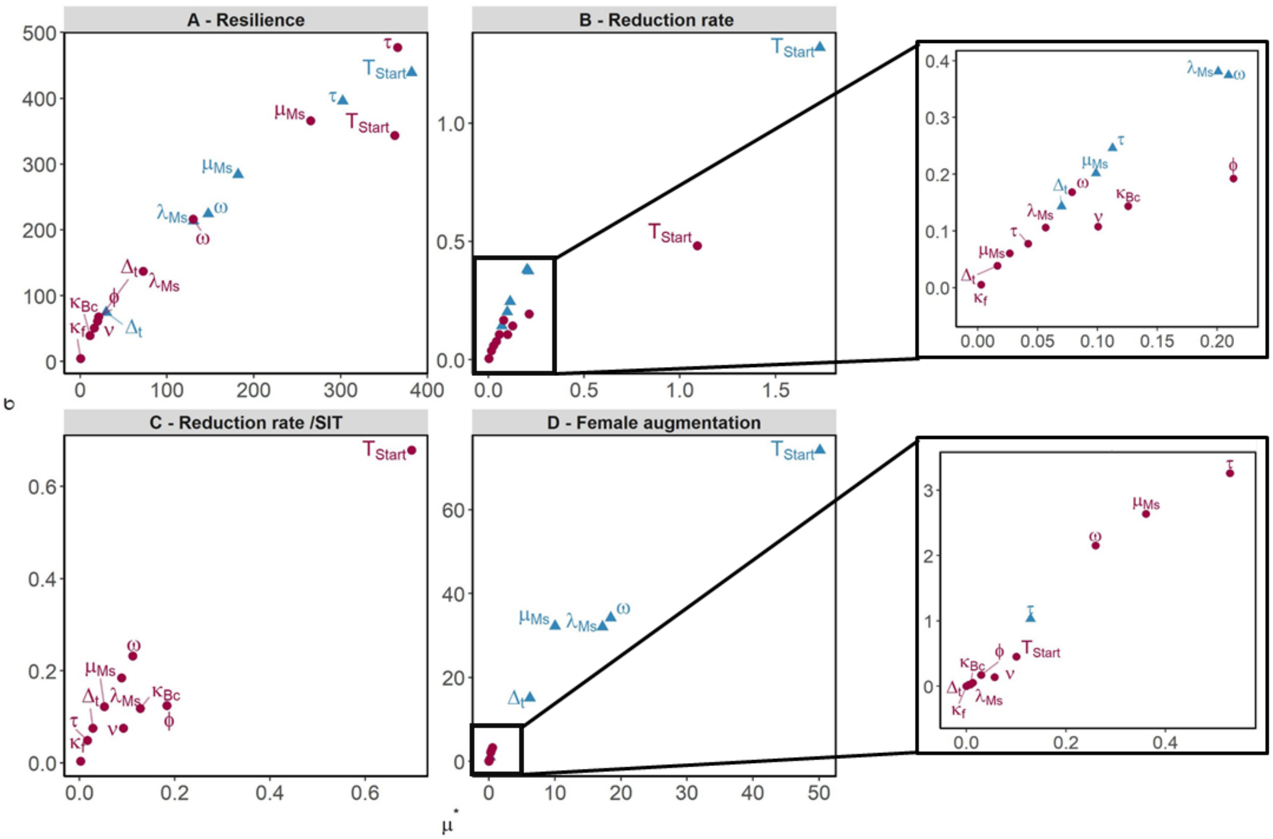

Figure 5. Keys parameters of the SIT and BSIT in temperate climate. Sensitivity analysis performed on the SIT (blue triangles) and the BSIT (red points). Four outputs were studied: the resilience (A), the reduction rate (B), the reduction rate of the BSIT compared to the SIT (C), and the increase in the female population (D). The variance (σ) of each elementary effect is plotted against its absolute value (μ^*). Parameters without influence ($\sigma = 0$ and $\mu^* = 0$) were removed. The bottom-left region (low μ^* and σ) of the reduction rate (B) and the female population increase (D) plots have been magnified (black boxes).

For 3 outputs out of 4, the date for the start of the releases (T_{start}) is by far the most influencing factor (Figure 5 (B, C, D)). Its elementary effect absolute value, describing its overall influence on the outputs, is 2 to 100 times higher than the effect of other parameters. Its participation to the output has either a non-linear relationship with the value, or a strong interaction with other inputs (high σ). The length of the release (τ) and the sterile male mortality (μ_s) are parameters that can significantly

modify the population resilience after SIT or BSIT (Figure 5 (A)).

Even if the starting day of release remains by far the major influential parameter, BSIT is less sensitive than SIT to this parameter for the reduction rate and resilience outputs (Figure 5 (A, B)). The reduction rate of BSIT compared to SIT is mostly influenced by the starting day of releases, and, to a much lesser extent, by sterile males competitiveness (ω) and the number of sterile males released (λ_{M_s}) (Figure 5 (C)). Male competitiveness, starting date of releases and the quantity of sterile males released drive an undesirable augmentation of the females number in SIT, while none has been observed for BSIT (Figure 5 (D)). These parameters may act in interaction (high σ). Boost related parameters (*e.g.* the decontamination rate, ν, the larval resistance to PP, ϕ and the PP transfers, κ_{Bc} and κ_F) have few or no influence in the outputs (Figure 5).

References

1. Haramboure, M. *et al.* Modelling the control of Aedes albopictus mosquitoes based on sterile males release techniques in a tropical environment. *Ecol. Model.* **424**, 109002, DOI: 10.1016/j.ecolmodel.2020.109002 (2020).

2. White, S. M., Rohani, P. & Sait, S. M. Modelling pulsed releases for sterile insect techniques: fitness costs of sterile and transgenic males and the effects on mosquito dynamics. *J. Appl. Ecol.* **47**, 1329–1339, DOI: 10.1111/j.1365-2664.2010.01880.x (2010). WOS:000283983200020.

3. McIntire, K. M. & Juliano, S. A. How can mortality increase population size? A test of two mechanistic hypotheses. *Ecology* **99**, 1660–1670, DOI: 10.1002/ecy.2375 (2018). _eprint: https://esajournals.onlinelibrary.wiley.com/doi/pdf/10.1002/ecy.2375.

4. Neale, J. T. & Juliano, S. A. Finding the sweet spot: What levels of larval mortality lead to compensation or overcompensation in adult production? *Ecosphere* **10**, e02855, DOI: 10.1002/ecs2.2855 (2019). _eprint: https://esajournals.onlinelibrary.wiley.com/doi/pdf/10.1002/ecs2.2855.

5. Bossard, M., Feranec, J., Otahel, J. & others. CORINE land cover technical guide: Addendum 2000. Tech. Rep. (2000). Publisher: European Environment Agency Copenhagen.

6. Tran, A. *et al.* A Rainfall- and Temperature-Driven Abundance Model for Aedes albopictus Populations. *Int. J. Environ. Res. Public Heal.* **10**, 1698–1719, DOI: 10.3390/ijerph10051698 (2013).

7. Delatte, H., Gimonneau, G., Triboire, A. & Fontenille, D. Influence of temperature on immature development, survival, longevity, fecundity, and gonotrophic cycles of Aedes albopictus, vector of chikungunya and dengue in the Indian Ocean. *J. Med. Entomol.* **46**, 33–41 (2009).

8. Lacour, G. *et al.* Aedes albopictus (Skuse, 1894)(Diptera: Culicidae) in France: gonotrophic cycle, fecundity and longevity. In *Proceedings of the International Conference "Emerging Vector-borne Diseases in a Changing European Environment", Montpellier, France*, 10–12 (2010).

9. Lacour, G. *et al.* Winter is coming: diapause phenology, wing shape and size seasonality of a temperate population of Aedes albopictus (Diptera: Culicidae). In *Proceedings of the 18th European Society of Vector Ecology Conference, Montpellier, France*, 8–11 (2012).

10. Tran, A. *et al.* Complementarity of empirical and process-based approaches to modelling mosquito population dynamics with Aedes albopictus as an example—Application to the development of an operational mapping tool of vector populations. *PLOS ONE* **15**, e0227407, DOI: 10.1371/journal.pone.0227407 (2020).

11. Morris, M. D. Factorial Sampling Plans for Preliminary Computational Experiments. *Technometrics* **33**, 161–174, DOI: 10.1080/00401706.1991.10484804 (1991).
